# Design of a Soft Sensor Based on Long Short-Term Memory Artificial Neural Network (LSTM) for Wastewater Treatment Plants

**DOI:** 10.3390/s23229236

**Published:** 2023-11-17

**Authors:** Roxana Recio-Colmenares, Elizabeth León Becerril, Kelly Joel Gurubel Tun, Robin F. Conchas

**Affiliations:** 1 Environmental Technology Department, Centro de Investigación y Asistencia en Tecnología y Diseño del Estado de Jalisco, A.C., Av. Normalistas 800, Colinas de la Normal, Guadalajara 44270, Jalisco, Mexico; roxana.recio@academicos.udg.mx; 2 School of Engineering and Technological Innovation, University of Guadalajara, Campus Tonalá, Tonalá 45425, Jalisco, Mexico; 3 Electrical Engineering Department, Research Center and Advanced Studies of Instituto Politécnico Nacional (CINVESTAV), Unidad Guadalajara, Av. del Bosque 1145, El Bajío, Zapopan 45017, Jalisco, Mexico; robin.conchas@cinvestav.mx

**Keywords:** wastewater treatment, soft sensors, LSTM, quality index, organic substrates

## Abstract

Assessment of wastewater effluent quality in terms of physicochemical and microbial parameters is a difficult task; therefore, an online method which combines the variables and represents a final value as the quality index could be used as a useful management tool for decision makers. However, conventional measurement methods often have limitations, such as time-consuming processes and high associated costs, which hinder efficient and practical monitoring. Therefore, this study presents an approach that underscores the importance of using both short- and long-term memory networks (LSTM) to enhance monitoring capabilities within wastewater treatment plants (WWTPs). The use of LSTM networks for soft sensor design is presented as a promising solution for accurate variable estimation to quantify effluent quality using the total chemical oxygen demand (*TCOD*) quality index. For the realization of this work, we first generated a dataset that describes the behavior of the activated sludge system in discrete time. Then, we developed a deep LSTM network structure as a basis for formulating the LSTM-based soft sensor model. The results demonstrate that this structure produces high-precision predictions for the concentrations of soluble $X_{1}$ and solid $X_{2}$ substrates in the wastewater treatment system. After hyperparameter optimization, the predictive capacity of the proposed model is optimized, with average values of performance metrics, mean square error (MSE), coefficient of determination (R^2^), and mean absolute percentage error (MAPE), of 23.38, 0.97, and 1.31 for $X_{1}$, and 9.74, 0.93, and 1.89 for $X_{2}$, respectively. According to the results, the proposed LSTM-based soft sensor can be a valuable tool for determining effluent quality index in wastewater treatment systems.

## 1. Introduction

In nonlinear systems, such as biological ones, complex variables crucial for determining the quality of wastewater often prove challenging to measure in real time due to the presence of external disturbances and the nonlinear phenomena of these processes. Within this context, the importance lies in the design of digital sensors aimed at identifying variables hard to measure in biological processes, with a specific focus on wastewater treatment plants [1]. This approach plays an essential role in decision making for optimal operation of the process, offering practical and cost-effective alternatives to expensive or impractical conventional measurement devices. The implementation of these sensors not only brings economic benefits but also has a positive impact on the environment. In contrast to hardware sensors, digital detection techniques offer notable advantages, including delay-free estimation, low cost, simple maintenance, and high resistance to interferences [2]. Taking into account the modeling methodologies, digital sensor models can be classified into three groups: first principles models, data-based models, and hybrid models. First principles models are complex and require significant computational resources, making data-based models the preferred option. The latter incorporates a variety of techniques such as support vector regression (SVR), artificial neural networks (ANN), Bayesian regression learning (BRL), gaussian process regression (GPR), kernel ridge regression (KRR), Kalman filters (KF), partial least squares regression (PLS), and ensemble learning [3,4,5,6]. While they have proven effective in modeling complex processes in digital sensors [7,8], the main challenge remains of the handling of unlabeled data and model generalization. Conventional modeling methods for digital sensors are not ideal for addressing large datasets, unlabeled data, and extensive industrial samples, making it difficult to obtain stable and reliable results [9]. In recent years, the widespread use of deep learning has been crucial in various fields, such as speech recognition, computer vision, natural language processing, and bioinformatics. Pretrained deep neural networks have proven to be a promising solution in extracting latent variables, significantly improving adaptability compared to traditional methods [10,11]. Furthermore, the computational efficiency of digital sensors is crucial for their successful implementation in industrial environments [12,13]. In this context, LSTM neural network models are a promising approach for time series forecasting and prediction compared to other deep neural network structures, since LSTMs are specifically designed to handle sequences of data, making them suitable for time series prediction. They can capture long-term dependencies in the data, allowing them to model relationships over extended time horizons, which is often a challenge for traditional feed-forward neural networks (FFNNs). LSTMs can process sequences of varying lengths, adapting to the specific context of each sequence, while some other deep neural network models require fixed-length input. In contrast to traditional recurrent neural networks (RNNs) which can face the vanishing gradient problem, making it difficult for them to capture long-term dependencies, LSTMs are designed to mitigate this issue through their gating mechanisms, allowing for more stable training and improved long-term performance [14]. Additionally, LSTMs can effectively handle noisy data and are robust to variations in data quality, making them suitable for real-world scenarios. LSTMs have been successfully applied to a wide range of time series forecasting tasks, including weather forecasting [14], financial forecasting [15], stock price and energy consumption predictions [16]. Their versatility and performance have made them a popular choice in these domains. The importance of using LSTM neural networks compared to traditional techniques lies in their ability to effectively model and predict relevant states in complex systems. By leveraging their ability to capture long-term dependencies in the data and handle sequences of varying lengths, LSTMs overcome the limitations of traditional techniques, especially in the context of time series prediction in bioprocesses. The use of LSTMs offers a promising and robust solution for online prediction variables, which has significant implications for improving efficiency and performance in a wide range of industrial and wastewater treatment applications.

In this work, a LSTM-based soft sensor approach to predict substrate concentrations for evaluating the effluent quality in wastewater treatment plants is proposed. The selection of deep LSTM network architecture and the configuration of hyperparameters results from a systematic exploration of parameter values, guided by empirical experimentation and prior research in the field. It represents a trade-off between the solution quality of prediction generated by the LSTM model and computational efficiency tailored to our specific problem context.

## 2. Materials and Methods

### 2.1. Wastewater Treatment Plant Description

**Figure 1:**
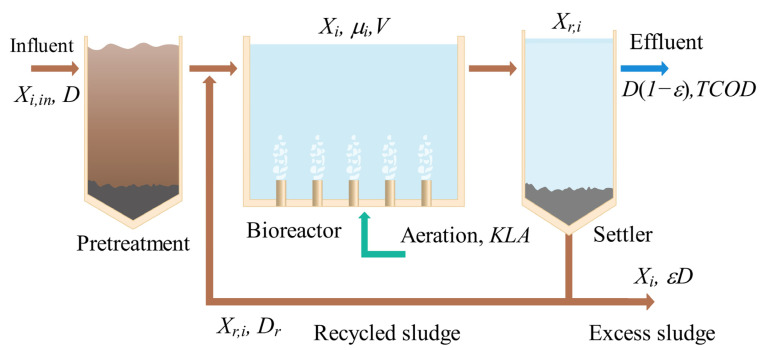
Wastewater plant configuration.

The treatment process is realized in a real small-sized plant consisting of an aeration tank with 2000 m^3^ as the working volume, mechanical aerators which provide oxygen (k_L_a = 4.5 h^−1^) and mix the incoming wastewater, and a settler for either solids to be recirculated to the aeration tank (*D*r) or extracted from the system (ε*D*). The influent average flow *D* is about 3000 m^3^/day, the average chemical oxygen demand input (*COD*in) is 320 mg/L, and the total nitrogen input (TNin) is 30 mg/L after pretreatment. The operational conditions used in this process are based on those given by [17]. The treatment plant is schematically demonstrated in Figure 1. The Activated Sludge Model (ASM1) is used to describe the biochemical transformation processes in the suspended-growth treatment reactor for chemical oxygen demand (*COD*) removal [18]. A reduced model is represented by Equations (1)–(6), composed of ordinary differential equations and nonlinear kinetic functions which bear resemblance to those explored in the studies referenced in [17,18]. The characterization of wastewater and estimation of parameter values were made according to [18], and the reduced model was validated in a previous work [19]. The fitted model provides a satisfactory understanding of the transformation process leading to *COD* removal. In this work, the data needed to train and test the LSTM network architectures used in the soft sensor model were generated by simulating the ASM1 reduced model. The main objective of wastewater treatment plants is to improve the effluent quality. Therefore, we quantify effluent quality using *TCOD* as the performance index. For example, for urban wastewater, the maximum specified concentration of *COD* leaving a small-sized wastewater treatment plant is *COD*_max_ = 150 mg/L [19]. The *TCOD* is given by Equation (1), composed of the easily biodegradable soluble substrate $X_{1,k}$, the slowly decomposing solid substrate component $X_{2,k}$, and the inert organic material $I_{s}$. The latter reflects the constant value of the inflow.

(1) $$TCOD=X_{1,k}+X_{2,k}+I_{s}$$

(2) $$X_{1,k+1}=D\left(X_{1,in}-X_{1,k}\right)-\frac{1}{YH}\cdot {\mu_{1}\cdot \mu }_{max,H}\left(\mu_{3}+\mu_{4}\cdot \mu_{6}\cdot \eta_{g}\right)X_{3,k}\;+\mu_{7}\cdot k_{h}\left(\mu_{3}+\mu_{4}\cdot \mu_{6}\cdot \eta_{h}\right)X_{3,k}$$

(3) $$X_{2,k+1}=D\left(X_{2,in}-X_{2,k}\right)+D_{r}\left(b-1\right)X_{2,k}+\left(1-f_{p}\right)\left(b_{H}\cdot X_{3_{k}}+b_{A}\cdot X_{B_{A}}\right)\;-\mu_{7}\cdot k_{h}\left(\mu_{3}+\mu_{4}\cdot \mu_{6}\cdot \eta_{h}\right)X_{3,k}$$

(4) $$X_{3,k+1}=D\left(X_{3,in}-X_{3,k}\right)+D_{r}\left(b-1\right)X_{3,k}+\mu_{1}\cdot \mu_{3}\cdot \mu_{max,H}\cdot X_{3,k}\;+\mu_{1}\cdot \mu_{4}\cdot \mu_{6}\cdot \mu_{max,H}\cdot \eta_{g}\cdot X_{3,k}-b_{H}\cdot X_{3,k}$$

(5) $$X_{4,k+1}=D\left(X_{4,in}-X_{4,k}\right)+D_{r}\left(b-1\right)X_{4,k}+\mu_{2}\cdot \mu_{5}\cdot \mu_{max,A}\cdot X_{4,k}-b_{A}X_{4,k}$$

(6) $$X_{5,k+1}=D\left(X_{5,in}-X_{5,k}\right)+KLA\left(X_{5,max}-X_{5,k}\right)-\left(\frac{1-Y_{H}}{Y_{H}}\right)\cdot \mu_{1}\cdot \mu_{3}\cdot \mu_{max,H}\cdot X_{3,k}\;-\left(\frac{4.57-Y_{A}}{Y_{A}}\right)\mu_{2}\cdot \mu_{5}\cdot \mu_{max,A}\cdot X_{4,k}$$

where $X_{3,k}$ is the active heterotrophic particulate biomass, $X_{4,k}$ is the active autotrophic particulate biomass, and $X_{5,k}$ is soluble oxygen. The kinetic and stoichiometric parameters are detailed in Appendix A, Table A1 and Table A2, respectively.

### 2.2. LSTM Network Architecture

**Figure 2:**
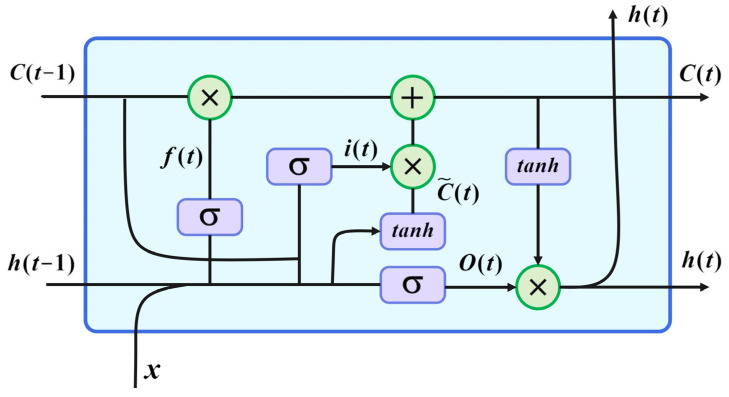
LSTM cell.

The LSTM is a type of recurrent neural network initially introduced in the field of deep learning by Hochreiter and Schmidhuber [20] to address the issue of gradient explosion in RNNs during backpropagation. The LSTM model is widely recognized as an influential architecture for learning from sequential data due to its ability to capture long-term dependencies and effectively learn from sequences of varying lengths. A schematic of the LSTM model unit is presented in Figure 2.

The LSTM unit comprises three gates responsible for controlling the flow of information: $\left(i\right)$ the input gate, which determines the significance of input information to be remembered; $\left(ii\right)$ the forget gate, which decides whether to retain or discard the input value; and $\left(iii\right)$ the output gate, which governs the output of the LSTM unit. LSTM is implemented through Equations (7)–(12).

The input gate $\left(i\left(t\right)\right):$

(7) $$i\left(t\right)=\sigma \left(W_{xi}x\left(t\right)+W_{hi}h\left(t\;1\right)+W_{ci}C\left(t\;1\right)+b_{i}\right)$$

The forget gate $\left(\;f\;\left(t\right)\right)$:

(8) $$f\left(t\right)=\sigma \left(W_{xf}x\left(t\right)+W_{hf}h\left(t-1\right)+W_{cf}C\left(t-1\right)+b_{f}\right)$$

The state candidates $\left(\tilde{C}\left(t\right)\right)$:

(9) $$\tilde{C}\left(t\right)=tanhtanh\;\left(W_{xc}x\left(t\right)+W_{hc}h\left(t-1\right)+b_{c}\right)\;$$

The activation cell $\left(C\left(t\right)\right)$:

(10) $$C\left(t\right)=f\left(t\right)C\left(t-1\right)+i\left(t\right)\tilde{C}\left(t\right)$$

The output gate $\left(o\left(t\right)\right)$:

(11) $$o\left(t\right)=\sigma \left(W_{xo}x\left(t\right)+W_{ho}h\left(t-1\right)+W_{co}C\left(t\right)+b_{o}\right)$$

The hidden state $\left(h\left(t\right)\right)$:

(12) $$h\left(t\right)=o\left(t\right)tanh\left(C\left(t\right)\right)\;$$

Regarding the components entailed in the mathematical depiction of the LSTM cell, $W_{ci}$, $W_{cf}$, and $W_{co}$ represent the weights establishing connections between the activation cell and the input gate, the forget gate, and the output gate, respectively. $W_{hi}$*,* $W_{hf}$*,* $W_{hc}$*,* and $W_{ho}$ denote the weights linking the hidden layer to the input gate, the forget gate, the activation cell, and the output gate [13]. Additionally, $W_{xi}$*,* $W_{xf}$*,* $W_{xc}$, and $W_{xo}$ correspond to the weight matrices connecting the input layer to the input gate, whereas $b_{i}$*,* $b_{f}$*,* $b_{c}$, and bo refer to the respective biases. Ultimately, the values are rescaled within the range of −1 to 1 using the tanh activation function.

### 2.3. LSTM-Based Soft Sensor Model

**Figure 3:**
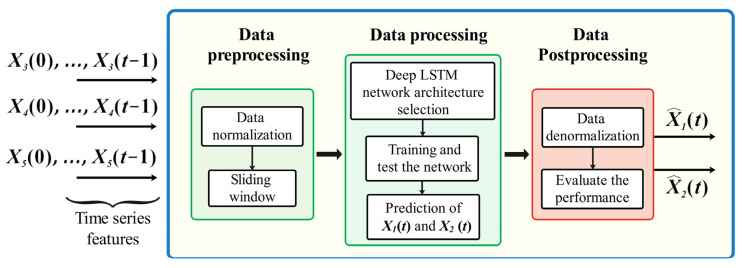
LSTM-based soft sensor.

In recent years, soft sensors, which estimate process variables using measured data from other sensors, have become increasingly popular due to their ability to provide accurate and reliable predictions. In this context, ANNs have emerged as a prominent approach for developing soft sensors due to their ability to handle complex nonlinear relationships and their capability to learn from data [21,22]. In this work, a deep LSTM network is chosen for modeling the temporal behavior and dependencies between WWTP inputs and outputs due to its capability for time series prediction and handling time-dependent values [23,24]. Thus, the proposed LSTM-based soft sensor model is responsible for predicting the $X_{1}$ and $X_{2}$ states to quantify effluent quality using the TCOD as the quality index. As shown in Figure 3, the model operates in three stages:

- Data preprocessing: this step includes data normalization and implementing a sliding window into the dataset.
- Data processing: this step comprises the selection, training, and testing of the deep LSTM network to predict $X_{1}$ and $X_{2}$.
- Data postprocessing: this step consists of the denormalization of data and the evaluation of the model’s performance, resulting in the predictions of $X_{1}$ and $X_{2}$, denoted as ${\hat{X}}_{1}$ and ${\hat{X}}_{2}$, respectively.

The $X_{3}$, $X_{4}$, and $X_{5}$ states are the input data measurements because of their role in the biotransformation of organic micropollutants (OMPs). Table 1 presents the input and output measurements of the proposed LSTM soft sensor.

### 2.4. Dataset and Data Processing

Preparing data before feeding it into a model is a crucial step in machine learning techniques. LSTM networks require sufficient historical information to predict future outcomes and enhance system performance. In this study, wastewater dynamic states $X_{3}$, $X_{4}$, and $X_{5}$ are considered as input parameters. Input parameters are assumed to be available for data acquisition and they are directly related to the substrates degradation and oxidation, so they are suitable for the identification of organic substrates. Simultaneously, the output parameters $X_{1}$ and $X_{2}$ are predicted by the LSTM-based soft sensor model to determine the TCOD quality index for wastewater effluent assessment. Based on the simplified WWTP model described by Equations (1)–(6), a dataset comprising 5020 samples corresponding to 120 h of the process (5 days) was generated. The first 4500 rows corresponding to the first 108 h of the process were used for training and validating the LSTM networks. After adjusting the hyperparameters and attaining the optimal results, the remaining 520 sets of data (4501–5020) from the dataset, representing the final 12 h of the process, were used as unseen data to forecast the levels of $X_{1}$ and $X_{2}$.

**Figure 4:**
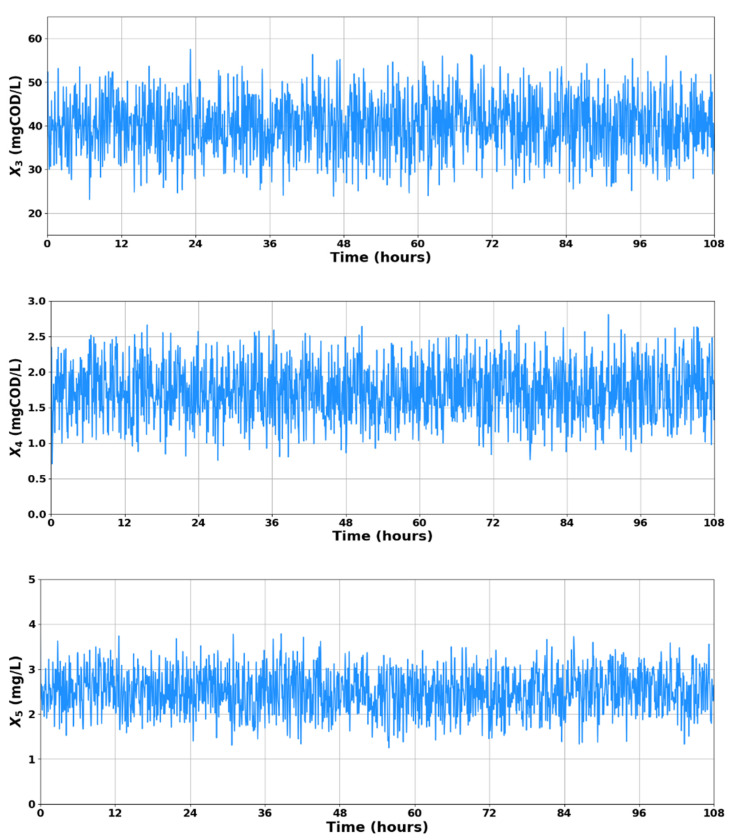
Dataset of input measurements *X*_3_, *X*_4_, and *X*_5_.

During the training phase, the model underwent supervised learning with predefined target outcomes. In the testing phase, the developed model was applied to predict the targeted substances based on the training data. Figure 4 visually demonstrates the 4500 data points generated for $X_{3}$, $X_{4}$, and $X_{5}$. Statistics of parameters of the variables in the dataset generated experimentally by employing the model described by Equations (1)–(6) are presented in Table 2. It is important to note that for all kinds of data-driven models (e.g., artificial intelligence-based models), a low standard deviation of data indicates that the data points are closely clustered around the mean, which implies a smaller degree of variability or dispersion in the data; thus, it is expected to get less biased outputs from the models [25].

**Figure 5:**
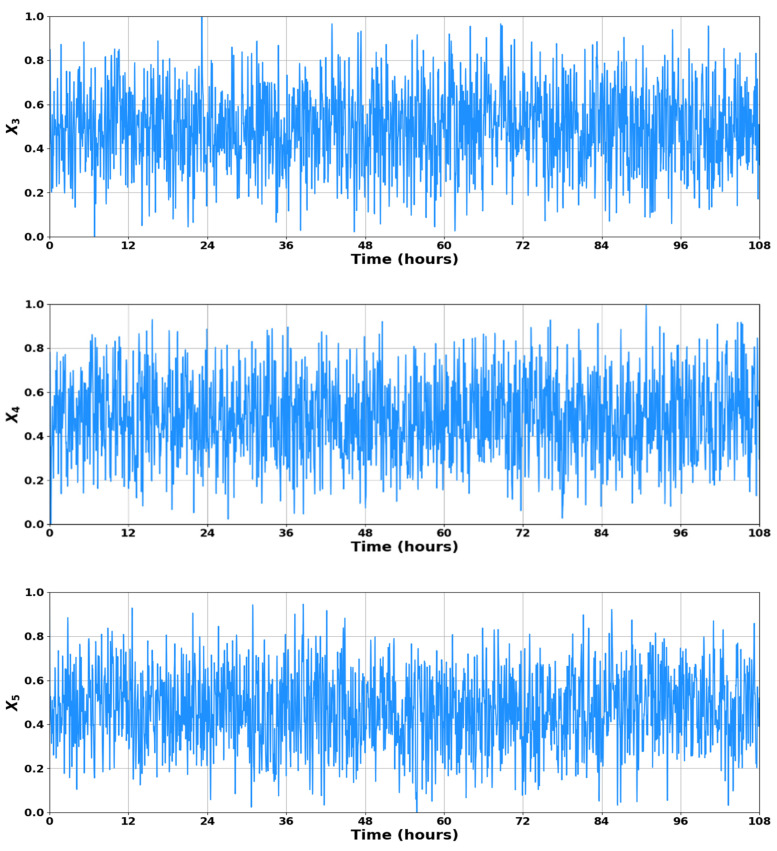
Normalized dataset of input measurements *X*_3_, *X*_4_, and *X*_5_.

Studies have suggested that LSTM networks are responsive to dataset randomization, particularly when utilizing nonlinear activation functions. A widely adopted strategy to address this challenge is normalizing the dataset within the 0 to 1 range [24]. Consequently, we standardized both the input and target datasets using systematic weight initialization to expedite the learning process, leading to quicker convergence. The final normalized input data used for training the LSTM networks is illustrated in Figure 5.

### 2.5. Hyperparameter Selection for Proposed LSTM Architecture

**Figure 6:**
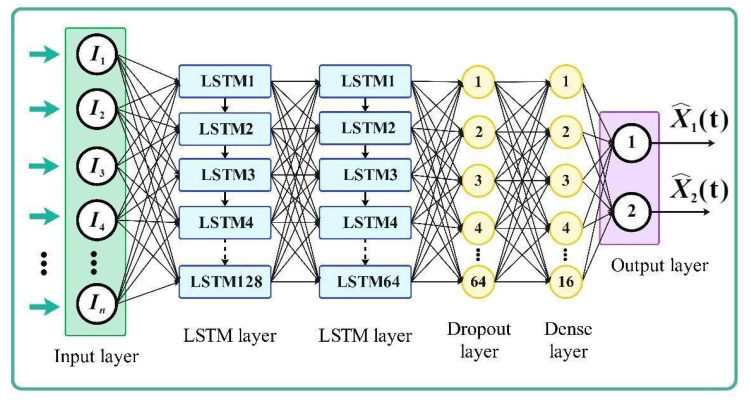
Proposed deep LSTM network architecture.

The adequate selection of the deep LSTM network architecture, which is the core of the LSTM-based soft sensor model presented in Figure 6, involves utilizing various tools and methodologies. The optimal number of LSTM units in the hidden layer is determined through systematic experimentation, ranging from 2 to 200 cell units. Each topology is tested using a loss function as the error metric, with the process repeated thrice to ensure result consistency. After careful experimentation, the deep LSTM network architecture, depicted in Figure 6, exhibited the best training and validation accuracy results.

The training process involved using the seven most recent past measurements to perform the prediction of substrates. Notable minor hyperparameters of the selected configuration are presented in Table 3. The proposed LSTM network was implemented in Python 3.10.12 software, utilizing the Keras library with TensorFlow as its backend framework. Table 4 lists various available open-source libraries employed in this study.

### 2.6. Model Performance Evaluation

The objective of model performance evaluation is to validate the accuracy of the proposed model and identify any errors, thus guaranteeing its reliable applicability [15]. In this study, we employ the MSE, R^2^, and MAPE as three performance metrics to evaluate the predictive capabilities of the proposed LSTM-based soft sensor model. The calculations for MSE, $R^{2}$, and MAPE are as follows [29]:

1. MSE: it measures the average of the squares of the errors and is given by the following equation:

(13) $$MSE=\frac{1}{n}\sum_{i=1}^{n}{\left(y_{i}-{\hat{y}}_{i}\right)}^{2}$$

2. R^2^: The coefficient of determination measures how much one variable can explain the variation in another variable when predicting the outcome of an event. The formula is as follows:

(14) $$R^{2}=1-\frac{\sum_{i=1}^{n}{\left(y_{i}-{\hat{y}}_{i}\right)}^{2}}{{\sum_{i=1}^{n}\left(y_{i}-{\underline{\hat{y}}}_{i}\right)}^{2}}$$

3. MAPE: it is the mean or average of the absolute percentage errors of prediction:

(15) $$MAPE=\frac{1}{n}\sum_{i=1}^{n}\left|\frac{y_{i}-{\hat{y}}_{i}}{{\hat{y}}_{i}}\right|\times 100$$

Regarding Equations (13)–(15), n represents the number of samples, $y_{i}$ corresponds to the i-th sample of the observed output data, ${\hat{y}}_{i}$ is the i-th predicted value, and ${\underline{\hat{y}}}_{i}$ is the mean of the predicted values.

## 3. Prediction Results

### 3.1. Training and Validation Stage

**Figure 7:**
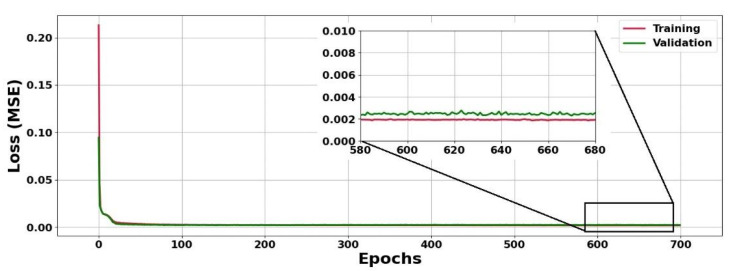
Loss curves of training and validation of proposed deep LSTM network.

Before running the proposed deep LSTM neural network for $X_{1}$ and $X_{2}$ prediction in the WWTP model described by Equations (1)–(6), the generated dataset comprising 5020 samples was divided into three groups, including data for training, data for validation, and data for testing, respectively. As mentioned in the previous section, a group of data comprised of 4500 rows corresponding to the first 108 h of the process was used for training and validating the LSTM networks, of which 80% was employed for training, and the remaining 20% of data was employed for validation. Figure 7 shows the training loss and validation loss curves in terms of MSE for the developed deep LSTM network using the hyperparameters presented in Table 3. From Figure 7, it is possible to observe that no overfitting occurs during the training and validation of the LSTM network stage.

### 3.2. Testing Stage

**Figure 8:**
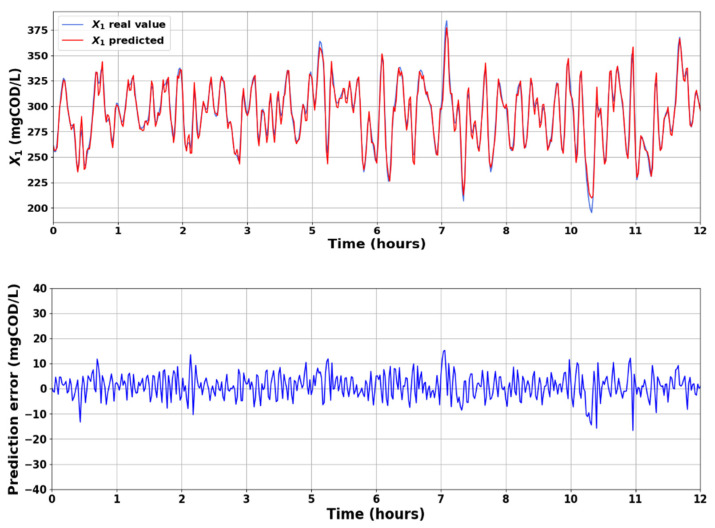
Observed and predicted results for *X*_1_.

**Figure 9:**
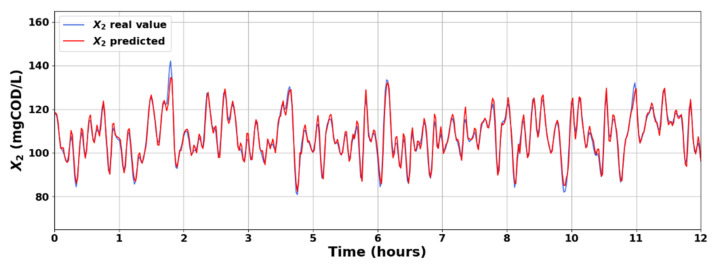

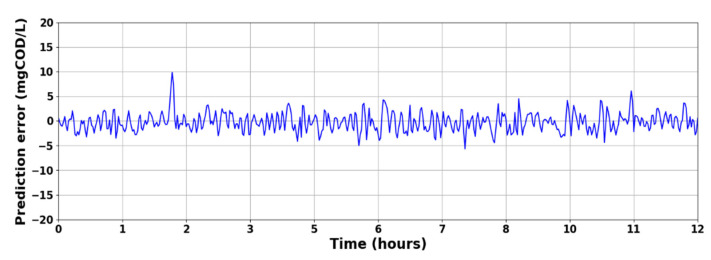
Observed and predicted results for *X*_2_.

**Figure 10:**
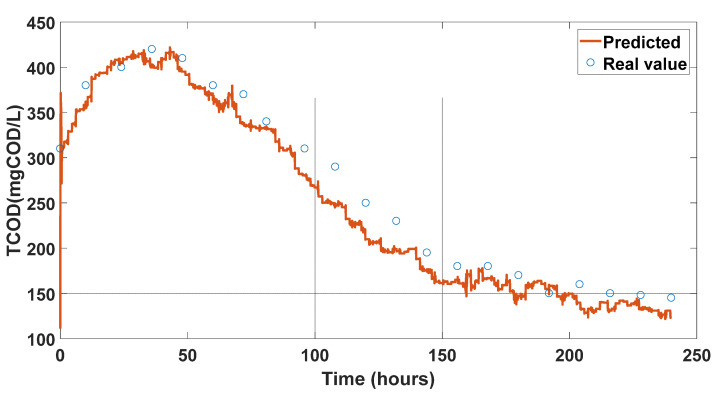
TCOD predicted vs. real values.

After training and validating the deep LSTM network, we applied the testing data containing 520 rows to evaluate the model’s prediction reliability for unseen data during the training process. Figure 8 shows the prediction results for $X_{1}$ with the respective prediction error. The prediction results for $X_{2}$ are presented in Figure 9. The states of the system are available via the LSTM-based soft sensor model, and the TCOD quality index is calculated by Equation (1). Figure 10 displays the predicted TCOD vs the real value in the wastewater plant along 240 h. From these results, it can be appreciated that, in general, the predicted values were close to the observed values, indicating the adequate capability of the proposed strategy to predict the behavior of $X_{1}$ and $X_{2}$ for unseen data.

The prediction results of the proposed model were evaluated based on the performance metrics MSE, $R^{2}$, and MAPE presented in Equations (13)–(15). Generally speaking, a good fit between the observed and predicted results is obtaining values of MSE close to zero, while obtaining values of $R^{2}$ close to 1. Table 5 presents a comparative analysis in terms of the performance metrics for the prediction results achieved by employing the proposed LSTM-based model against those achieved using the FFNN [23] technique. The comparison was conducted using the same test dataset for both techniques. The FFNN was implemented with a single hidden layer comprising 128 neurons and trained using the Levenberg–Marquardt Algorithm (LMA).

**Figure 11:**
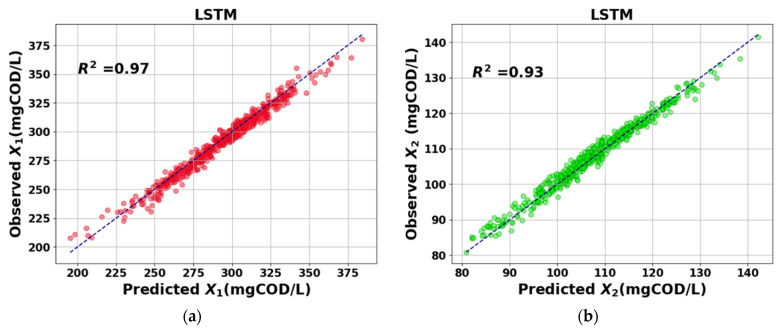
Plot of observed versus predicted values and the fitted regression line using the LSTM-based soft sensor model. (**a**) Red circles indicate *X*_1_ positive correlation and (**b**) green circles indicate *X*_2_ positive correlation.

**Figure 12:**
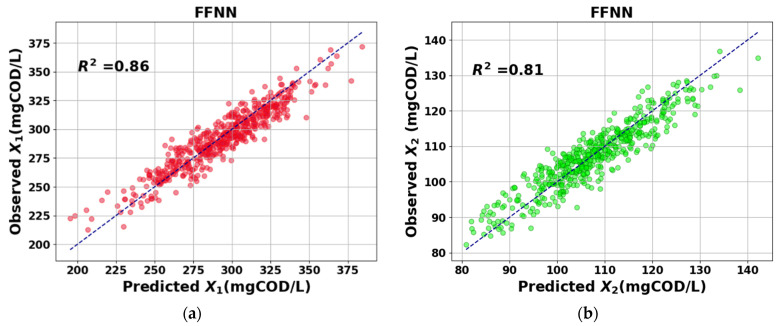
Plot of observed versus predicted values and the fitted regression line using the FFNN. (**a**) Red circles indicate *X*_1_ positive correlation and (**b**) green circles indicate *X*_2_ positive correlation.

Marquardt algorithm. The average MSE, $R^{2}$, and MAPE values obtained were 23.38, 0.97, and 1.31 for $X_{1}$, and 9.74, 0.93, and 1.89 for $X_{2}$, respectively. The results indicate a superior performance of the proposed approach over the results obtained using the FFNN. Figure 11 and Figure 12 show the scatter plot of the real versus predicted values of $X_{1}$ and $X_{2}$ for the LSTM-based model and FFNN technique, respectively.

## 4. Discussion

Based on the results presented in Table 5, it can be observed that the MSE for $X_{2}$ prediction was comparatively better than that for $X_{1}$, which can be attributed to some large punctual deviations due to outliers in the dataset. Hence, some of the peaks or differences shown in the plots could be attributed to these outliers, leading to substantial deviations in the consecutive results. This complication could be addressed with a more extensive preprocessing process of the dataset. On the other hand, the prediction results in terms of performance metrics $R^{2}$ and MAPE were better for $X_{1}$ in contrast to the prediction results for $X_{2}$. These obtained results can be attributed to the fact that according to the basic statics of variables in the dataset presented in Table 3, the easily biodegradable soluble substrate $X_{1}$ presents a lower value of standard deviation, which implies a low data variability and a more stable and predictable pattern, resulting in more accurate and less biased predictions generated by the model. In general, the results demonstrate that the proposed LSTM-based soft sensor model is competent in capturing the nonlinear behavior of substrates $X_{1}$ and $X_{2}$ present in the wastewater biological process for effluent quality evaluation.

## 5. Conclusions

This study proposes an LSTM-based soft sensor model to predict the concentrations of two critical substrates for effluent quality determination in wastewater treatment plants. First, we generated a dataset that describes the behavior of a real small-sized WWTP, modeled by the discrete-time ASM1. Then, we developed a deep LSTM network structure as the foundation for formulating the LSTM-based soft sensor model. The results demonstrate that this structure yields high-accuracy predictions for the organic substrates. After hyperparameter fine-tuning, the predictive capability of the proposed model was optimized, with average values of the performance metrics MSE, $R^{2}$, and MAPE of 23.38, 0.97, and 1.31 for substrate $X_{1}$, and 9.74, 0.93, and 1.89 for substrate $X_{2}$, respectively. According to the results, the proposed LSTM-based soft sensor can be a valuable management tool for decision making, with the aim to satisfy legislative requirements. However, it is important to note that LSTM networks still present several challenging limitations. For instance, LSTMs are prone to overfitting when dealing with small datasets. Additionally, data preparation is critical for LSTM predictions. In most cases, it is necessary to normalize or standardize the data, handle missing values, and select appropriate features to ensure that the LSTM can effectively learn from the input. Therefore, at this time, the authors are actively exploring the development of methodologies for optimally selecting the most suitable LSTM network structure and the respective hyperparameters according to the particular application. Furthermore, as future work, it is intended to investigate the application of the proposed LSTM-based soft sensor to simulate a closed-loop wastewater treatment plant system.

## Figures and Tables

**Table 1:** Input and output measurements of LSTM soft sensor.

Input Measurements	
Measurement	Description
$X_{3}(mgCOD/L)$	Active heterotrophic particulate biomass
$X_{4}(mgCOD/L)$	Active autotrophic particulate biomass
$X_{5}(mg/L)$	Soluble oxygen
**Output measurements**	
$X_{1}(mgCOD/L)$	Easily biodegradable soluble substrate
$X_{2}(mgCOD/L)$	Slowly biodegradable particulate substrate

**Table 2:** Basic statistics of parameters in the generated dataset.

Parameters	Minimum	Maximum	Mean	Std. Deviation
$X_{1}(mgCOD/L)$	189.13	393.60	290.82	31.77
$X_{2}(mgCOD/L)$	66.06	145.53	107.80	11.91
$X_{3}(mgCOD/L)$	23.10	57.54	40.13	5.84
$X_{4}(mgCOD/L)$	0.70	2.80	1.73	0.35
$X_{5}(mg/L)$	1.24	3.93	2.49	0.43

**Table 3:** Hyperparameters selected for the deep LSTM architecture.

Hyperparameters	Selected Values
Batch size	128
Previous time steps	7
Optimizer	Adam
Epoch size	700
Dropout rate	0.1
Optimizer learning rate	0.001
Number LSTM layers	2
Number of LSTM cells per layer	$L_{1}$ 128 and $L_{2}$ 64
Activation function	RELU

**Table 4:** Libraries of Python 3.10.12 employed in this work.

Library	Purpose	Version
Numpy [25]	Data processing	1.23.5
Pandas [26]	Data management	1.5.3
Matplotlib [27]	Graphic Generation	3.7.1
Tensorflow [28]	Neural network implementation	2.13.0

**Table 5:** Summary of prediction performance.

Method	Metric of Performance	Obtained Values $\mathrm{for}\;X_{1}$	Obtained Values $\mathrm{for}\;X_{2}$
Proposed LSTM-based approach	MSE	23.38	9.74
	$R^{2}$	0.97	0.93
	MAPE (%)	1.31	1.89
FFNN	MSE	115.52	13.70
	$R^{2}$	0.86	0.81
	MAPE (%)	3.34	2.76

## Data Availability

Data are contained within the article.

## Appendix A

**Table A1:** Mathematical model parameters.

Parameter	Values	Units	Description
$Y_{A}\;$	0.24	mg COD/mg N	Autotrophic yield coefficient
$Y_{H}\;$	0.67	mg CDO/mg COD	Heterotrophic yield coefficient
$\mu_{max,A}\;$	0.8	1/h	Maximum specific growth rate for autotrophs
$\mu_{max,H}\;$	6	1/h	Maximum specific growth rate for heterotrophs
$\mu_{1}$	−	−	Monod kinetics for easily biodegradable soluble substrate
$\mu_{2}$	−	−	Monod kinetics for the component $X_{5,k}$ as a function of $X_{4,k}$
$\mu_{3}$	−	−	Monod kinetics for the component $X_{5,k}$ as a function of $X_{3,k}$
$\mu_{4}$	−	−	Monod kinetics for soluble nitrate and nitrite nitrogen
$\mu_{5}$	−	−	Monod kinetics for soluble ammonium nitrogen
$\mu_{6}$	−	−	Inhibition kinetics for $X_{5,k}$
$\mu_{7}$	−	−	Saturation kinetics
b	0.9	−	Fraction coefficient for $D_{r}$
$b_{A}\;$	0.05	1/h	Autotrophic decay coefficient
$b_{H}\;$	0.22	1/h	Heterotrophic decay coefficient
$f_{P}\;$	0.08	−	Fraction of biomass yielding particulate products
$\eta_{g}\;$	0.8	−	Correction factor for anoxic growth of heterotrophs

**Table A2:** Initial conditions and additional parameters.

Parameter	Values	Units	Description
D	2	1/h	Dilution rate
$D_{r}$	1	1/h	Dilution recycle rate
KLA	−	1/h	Oxygen transfer coefficient
$X_{i,\;in}$	200	mgCOD/L	Initial condition of $X_{1,k}$
$X_{2,in}$	100	mgCOD/L	Initial condition of $X_{2,k}$
$X_{3,in}$	0	mgCOD/L	Initial condition of $X_{3,k}$
$X_{4,in}$	0	mgCOD/L	Initial condition of $X_{4,k}$
$X_{5,in}$	2	mgCOD/L	Initial condition of $X_{5,k}$
$X_{5,max}$	10	mg/L	Maximum concentration of soluble oxygen
V	15	L	Tank volume
$I_{s}$	5	mgCOD/L	Concentration of soluble and particulate inert organic matter
$\eta_{h}$	0.4	-	Correction factor for anoxic hydrolysis
